# Supplementary material for: Advancing knock-in approaches for robust genome editing in zebrafish
Source: Biol Open. 2026 Feb 18;15(2):bio062472. doi: 10.1242/bio.062472 (PMC12958296; doi:10.1242/bio.062472)
Supplement: Supplementary information [file biolopen-15-062472-s1.pdf]

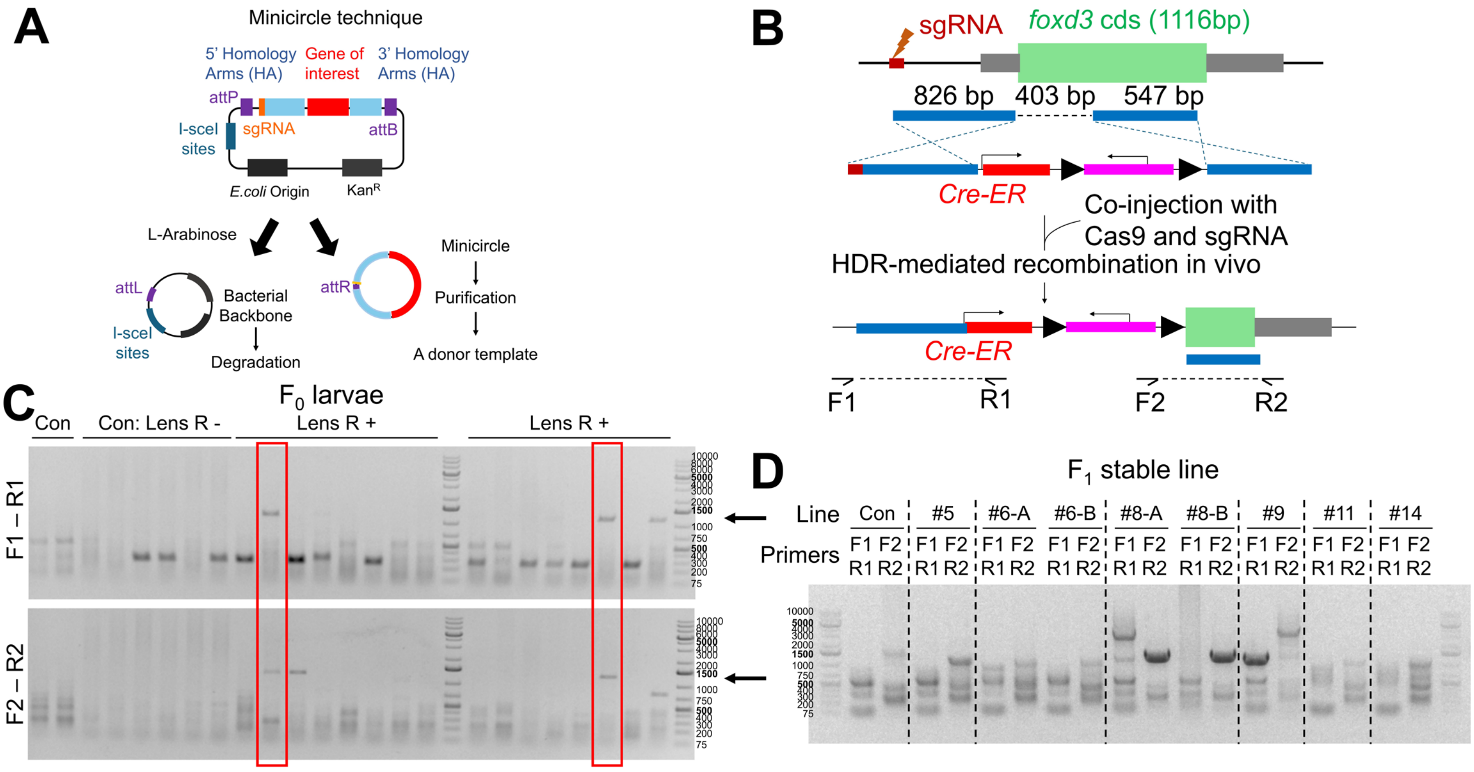

Fig. S1. ***foxd3 CreER* knock-in reporter generation.** (**A**) Schematic of minicircle generation (**B**) Schema[illegible] of genome editing strategy to create *foxd3*^{CreER} KI line. (**C**) PCR genotyping of F_0 larvae targeting the upstream (F[illegible]1) and downstream (F2–R2) regions flanking the integration site at the *foxd3* locus. (**D**) PCR genotyping of F_1 stab[illegible]ne targeting the upstream (F1–R1) and downstream (F2–R2) regions flanking the integration site at the *foxd3* locus.

Biology Open • Supplementary information

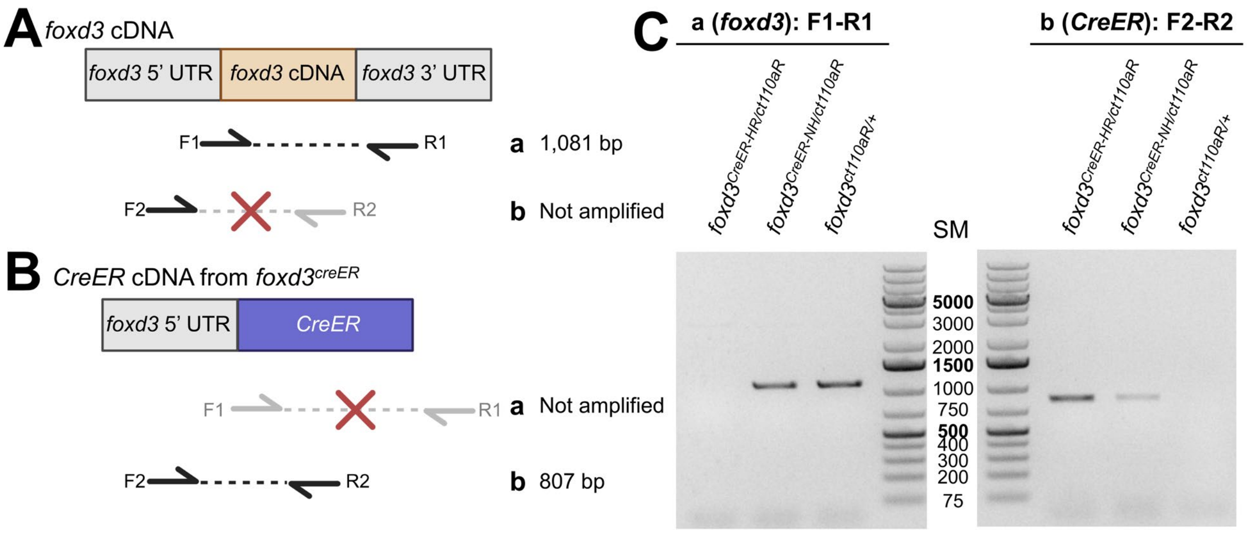

Supplementary Fig. S2. *foxd3*$^{CreER\text{-}HR}$ lacks *foxd3* expression. (**A, B**) cDNA structure of *foxd3* (**A**) and *CreER* of *foxd3*^{CreER} KI lines (**B**). (**C**) RT-PCR analysis indicates lack of *foxd3* cDNA expression in *foxd3*$^{CreER\text{-}HR}$, but not *foxd3*$^{CreER\text{-}NH}$ (primer set #a). *CreER* cDNA is detectable in both *foxd3*$^{CreER\text{-}NH}$ and *foxd3*$^{CreER\text{-}HR}$ lines (primer set #b).

Biology Open • Supplementary information

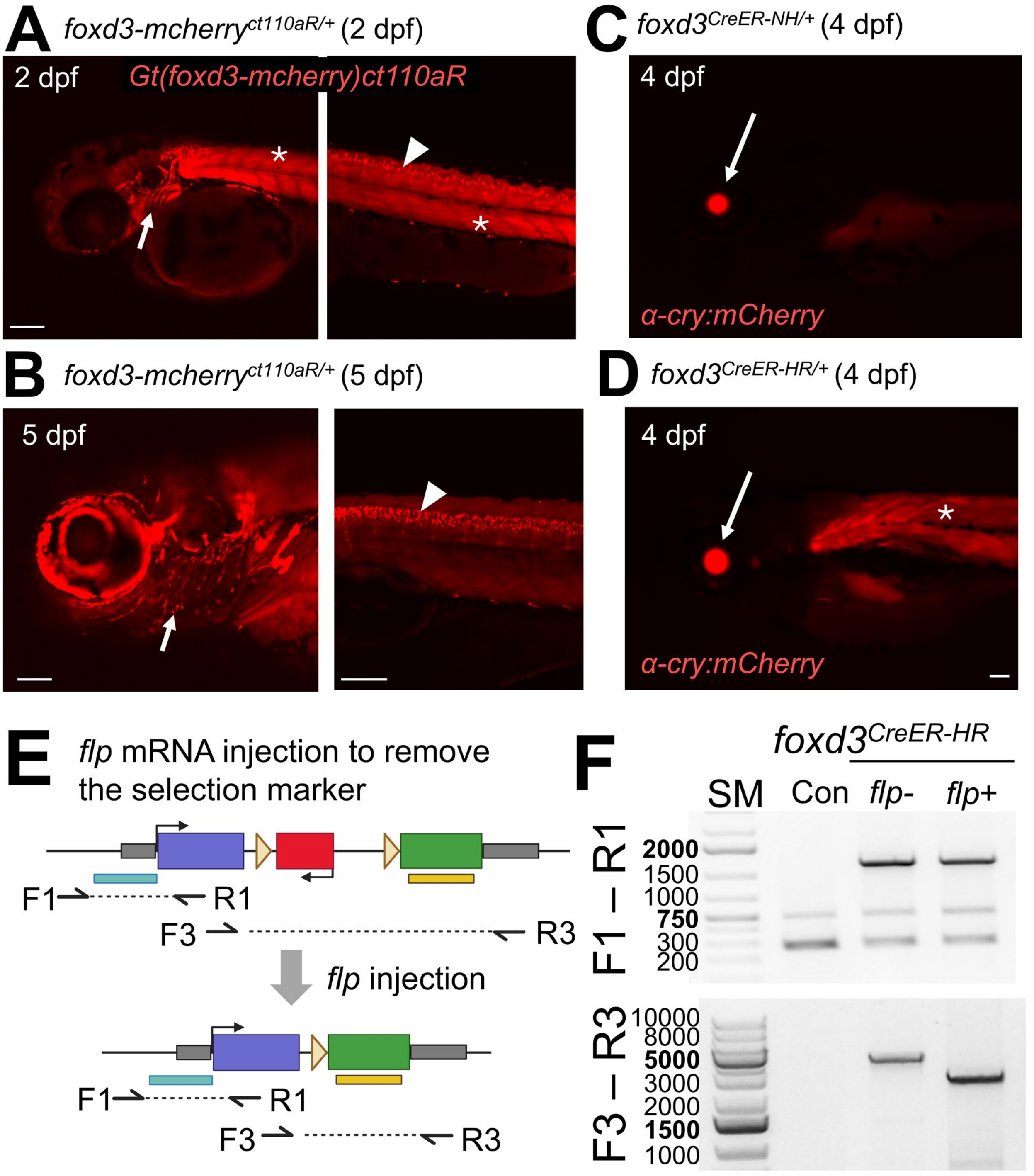

Biology Open • Supplementary information

(asterisks). Expression in the paraxial mesoderm declines by 5 dpf. (**C, D**) Expression pattern of the *α-cry:mCherry* selection marker in *foxd3*$^{CreER\text{-}NH/+}$ (**C**) and *foxd3*$^{CreER\text{-}HR/+}$ (**D**). While lens expression in observed in both lines (arrows), additional paraxial mesoderm expression is found in *foxd3*$^{CreER\text{-}HR/+}$ (asterisk). (**E**) Schematic of removing *FRT*-franked *α-cry:mCherry* selection marker cassette by *flp* mRNA injection (**F**) PCR genotyping of *flp*-injected larvae confirmed the deletion of the selection marker. Primer target sites are shown in **E**.

Biology Open • Supplementary information

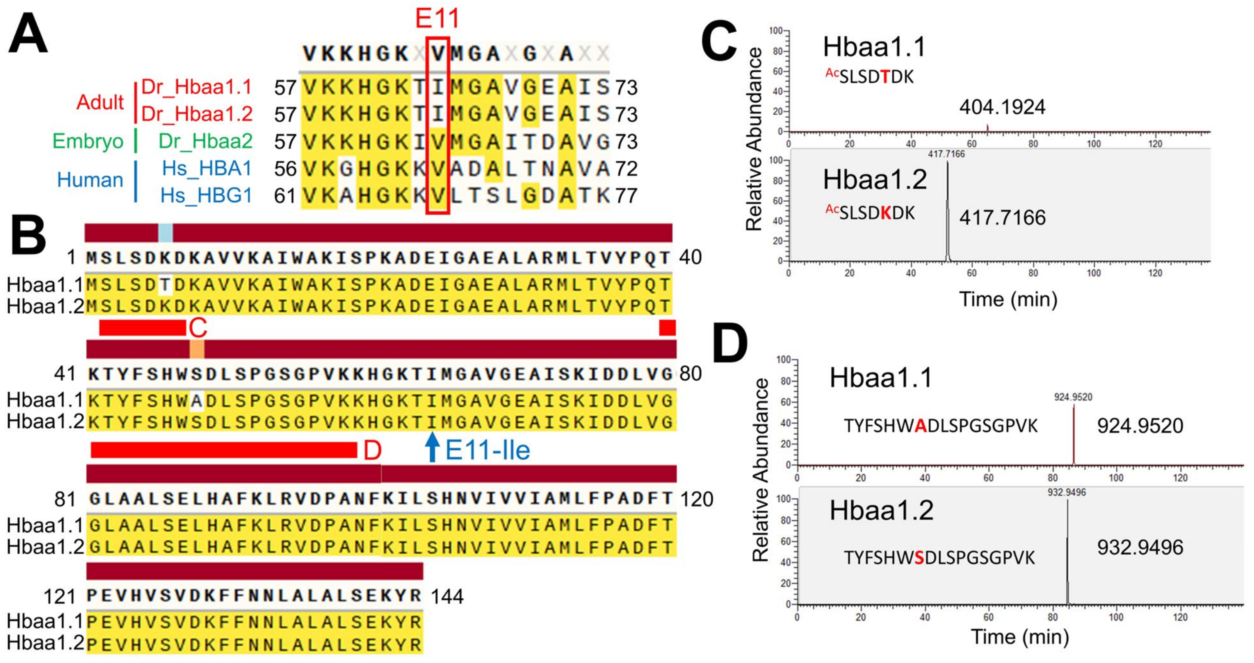

Fig. S4. Adult hemoglobin alpha genes in the zebrafish genome. (**A**) amino acid alignments near the targeted E11 region of adult and embryonic hemoglobin genes in zebrafish and humans. **(B)** a.a. sequence alignment between Hbaa1.1 and Hbaa1.2 reveals two a.a. differences. (**C, D**) Mass spectrometry analysis quantifying peptides derived from Hbaa1.1 and Hbaa1.2 in adult zebrafish blood. The unique peptide sequences used for quantification are indicated in (**B**).

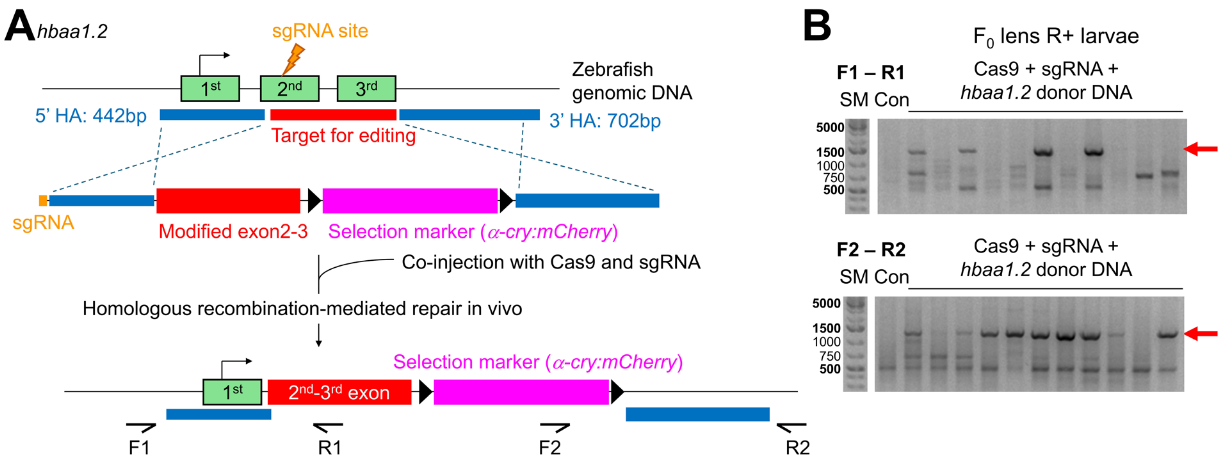

Fig. S5. Genome engineering to substitute I to V in Hbaa1.2. (**A**) Schematic of genome editing strategy to create *hbaa1.2*^{ItoV} allele. (**B**) PCR genotyping of F_0 larvae targeting the upstream (F1–R1) and downstream (F2–R2) regions flanking the integration site at the *hbaa1.2* locus.

Biology Open • Supplementary information

Table S1. Plasmid list of the mini-golden

Plasmid Name	Addgene Plasmid ID	Linker between BsaI
pMC-Dest-BsaI	200550	CAGT-CCAA
pMC-ME-v1_EGFP-pA	200513	AGTC-CTCA
pMC-ME-v1_2a-EGFP-pA ORF-1 with ATG	200514	AGTC-CTCA
pMC-ME-v1_2a-EGFP-pA ORF-2 with ATG	200515	AGTC-CTCA
pMC-ME-v1_2a-EGFP-pA ORF-3 with ATG	200516	AGTC-CTCA
pMC-ME-v1_2a-EGFP-pA ORF-1 without ATG	200517	AGTC-CTCA
pMC-ME-v1_2a-EGFP-pA ORF-2 without ATG	200518	AGTC-CTCA
pMC-ME-v1_2a-EGFP-pA ORF-3 without ATG	200519	AGTC-CTCA
pMC-ME-v1_memb-EGFP-pA	200520	AGTC-CTCA
pMC-ME-v1_mCherry-pA	200521	AGTC-CTCA
pMC-ME-v1_2a-mCherry-pA ORF-1 with ATG	200522	AGTC-CTCA
pMC-ME-v1_2a-mCherry-pA ORF-2 with ATG	200523	AGTC-CTCA
pMC-ME-v1_2a-mCherry-pA ORF-3 with ATG	200524	AGTC-CTCA
pMC-ME-v1_2a-mCherry-pA ORF-1 without ATG	200525	AGTC-CTCA
pMC-ME-v1_2a-mCherry-pA ORF-2 without ATG	200526	AGTC-CTCA
pMC-ME-v1_2a-mCherry-pA ORF-3 without ATG	200527	AGTC-CTCA
pMC-ME-v1_memb-mCherry-pA	200528	AGTC-CTCA
pMC-ME-v1_mCherry-P2A-NTRv2-pA	200529	AGTC-CTCA
pMC-ME-v1_T2A-mCherry-P2A-NTRv2-pA ORF-1	200530	AGTC-CTCA
pMC-ME-v1_T2A-mCherry-P2A-NTRv2-pA ORF-2	200531	AGTC-CTCA
pMC-ME-v1_T2A-mCherry-P2A-NTRv2-pA ORF-3	200532	AGTC-CTCA
pMC-ME-v1_BFP-pA	200533	AGTC-CTCA
pMC-ME-v1_2a-BFP-pA ORF-1 without ATG	200534	AGTC-CTCA
pMC-ME-v1_2a-BFP-pA ORF-2 without ATG	200535	AGTC-CTCA
pMC-ME-v1_2a-BFP-pA ORF-3 without ATG	200536	AGTC-CTCA
pMC-ME-v1_memb-BFP-pA	200537	AGTC-CTCA
pMC-ME-v1_BFP-P2A-NTRv2-pA	200538	AGTC-CTCA
pMC-ME-v1_T2A-BFP-P2A-NTRv2-pA ORF-1	200539	AGTC-CTCA
pMC-ME-v1_T2A-BFP-P2A-NTRv2-pA ORF-2	200540	AGTC-CTCA
pMC-ME-v1_T2A-BFP-P2A-NTRv2-pA ORF-3	200541	AGTC-CTCA
pMC-ME-v1_CreER-pA	200542	AGTC-CTCA
pMC-ME-v1_CreER-pA-FRT-acry:venus-pA-FRT	200543	AGTC-CTCA
pMC-ME-v1_CreER-pA-FRT-acry:mCherry-pA-FRT	200544	AGTC-CTCA
pMC-ME-v1_P2A-CreER-pA ORF1 FRT-acry:mCherry-pA-FRT	200545	AGTC-CTCA
pMC-ME-v1_P2A-CreER-pA ORF2 FRT-acry:mCherry-pA-FRT	200546	AGTC-CTCA
pMC-ME-v1_P2A-CreER-pA ORF3 FRT-acry:mCherry-pA-FRT	200547	AGTC-CTCA

Biology Open • Supplementary information

pMC-Dest-uni-gRNA5-BsaI	241150	CAGT-CCAA
pMC-ME-v2_EGFP-pA	241151	TCGC-CTCA
pMC-ME-v2_memb-EGFP-pA	241152	TCGC-CTCA
pMC-ME-v2_P2A-EGFP-pA-ORF-1withoutATG	241153	TCGC-CTCA
pMC-ME-v2_P2A-EGFP-pA-ORF-2withoutATG	241154	TCGC-CTCA
pMC-ME-v2_P2A-EGFP-pA-ORF-3withoutATG	241155	TCGC-CTCA
pMC-ME-v2_mCherry-pA	241156	TCGC-CTCA
pMC-ME-v2_memb-mCherry-pA	241157	TCGC-CTCA
pMC-ME-v2_P2A-mCherry-pA-ORF-1withoutATG	241158	TCGC-CTCA
pMC-ME-v2_P2A-mCherry-pA-ORF-2withoutATG	241159	TCGC-CTCA
pMC-ME-v2_P2A-mCherry-pA-ORF-3withoutATG	241160	TCGC-CTCA
pMC-ME-v2_memb-BFP-pA	241161	TCGC-CTCA
pMC-ME-v2_pMC-ME_2a-BFP-ORF-1 withoutATG	241162	TCGC-CTCA
pMC-ME-v2_P2A-BFP-pA-ORF-2withoutATG	241163	TCGC-CTCA
pMC-ME-v2_P2A-BFP-pA-ORF-3withoutATG	241164	TCGC-CTCA
pMC-ME-v2_CreER-pA_FRT-acrymCherry	241165	TCGC-CTCA
pMC-ME-v2_CreER-pA_FRT-acry-venus	241166	TCGC-CTCA
pMC-ME-v2_P2A-CreER-pA-ORF1-FRT-acrymCherry	241167	TCGC-CTCA
pMC-ME-v2_P2A-CreER-pA-ORF2-FRT-acrymCherry	241168	TCGC-CTCA
pMC-ME-v2_P2A-CreER-pA-ORF3-FRT-acrymCherry	241169	TCGC-CTCA
pMC-ME-v2_MCS_BsmBI	241170	TCGC-CTCA
pMC-ME-v2_mNeongreen	241171	TCGC-CTCA
pMC-ME-v2_linker-P2A-lifeact-mNeongreen-ORF-1	241172	TCGC-CTCA
pMC-ME-v2_linker-P2A-lifeact-mNeongreen-ORF-2	241173	TCGC-CTCA
pMC-ME-v2_linker-P2A-lifeact-mNeongreen-ORF-3	241174	TCGC-CTCA
pMC-ME-v2_memb-mNeongreen	241175	TCGC-CTCA
pMC-ME-v2_linker-P2A-mNeongreen-ORF1	241176	TCGC-CTCA
pMC-ME-v2_linker-P2A-mNeongreen-ORF2	241177	TCGC-CTCA
pMC-ME-v2_linker-P2A-mNeongreen-ORF3	241178	TCGC-CTCA
pMC-ME-v2_FRT-acrymCherry-FRT	241179	TCGC-CTCA
pMC-ME-v2_linker-T2A-NTRv2-linker-P2A-mNeonGreen ORF1	241180	TCGC-CTCA
pMC-ME-v2_linker-T2A-NTRv2-linker-P2A-mNeonGreen ORF2	241181	TCGC-CTCA
pMC-ME-v2_linker-T2A-NTRv2-linker-P2A-mNeonGreen ORF3	241182	TCGC-CTCA
pMC-ME-v2_linker-P2A-mem-mScarlet-ORF-1	241183	TCGC-CTCA
pMC-ME-v2_linker-P2A-mem-mScarlet-ORF-2	241184	TCGC-CTCA
pMC-ME-v2_linker-P2A-mem-mScarlet-ORF-3	241185	TCGC-CTCA
pMC-ME-v2_SA-linker-P2A-mem-mScarlet-ORF-1	241186	TCGC-CTCA
pMC-ME-v2_SA-linker-P2A-mem-mScarlet-ORF-2	241187	TCGC-CTCA
pMC-ME-v2_SA-linker-P2A-mem-mScarlet-ORF-3	241188	TCGC-CTCA

pMC-ME-v2_nzCas9n-GSG-linker-P2A-mNeongreen	241189	TCGC-CTCA
pMC-ME-v2_3x-FLAG-nzdCas9-V5-APEX2n-GSG-linker-P2A-mNeongreen	241190	TCGC-CTCA
pMC-ME-v2_nAPEX2-V5-zdCas9n-3x-FLAG-GSG-linker-P2A-mNeongreen	241191	TCGC-CTCA
pMC-ME-v2_linker-T2A-NTRv2-linker-P2A-mNeonGreen-CAAXORF1	241192	TCGC-CTCA
pMC-ME-v2_linker-T2A-NTRv2-linker-P2A-mNeonGreen-CAAXORF2	241193	TCGC-CTCA
pMC-ME-v2_linker-T2A-NTRv2-linker-P2A-mNeonGreen-CAAXORF3	241194	TCGC-CTCA
pMC-ME-v2_mCherry-P2A-NTRv2-pA	241195	TCGC-CTCA
pMC-ME-v2_T2A-mCherry-P2A-NTRv2-pA-ORF-1	241196	TCGC-CTCA
pMC-ME-v2_T2A-mCherry-P2A-NTRv2-pA-ORF-2	241197	TCGC-CTCA
pMC-ME-v2_T2A-mCherry-P2A-NTRv2-pA-ORF-3	241198	TCGC-CTCA
pMC-ME-v2_T2A-BFP-P2A-NTRv2-pA-ORF-1	241199	TCGC-CTCA
pMC-ME-v2_T2A-BFP-P2A-NTRv2-pA-ORF-2	241200	TCGC-CTCA
pMC-ME-v2_T2A-BFP-P2A-NTRv2-pA-ORF-3	241201	TCGC-CTCA
pMC-ME-v2_lifeact-mNeongreen-pA	241202	TCGC-CTCA
pMC-ME-v2_SA-TCGC-mNeongreen	241203	TCGC-CTCA
pMC-ME-v2_SA-linker-P2A-lifeact-mNeongreen-ORF-1	241204	TCGC-CTCA
pMC-ME-v2_SA-linker-P2A-lifeact-mNeongreen-ORF-2	241205	TCGC-CTCA
pMC-ME-v2_SA-linker-P2A-lifeact-mNeongreen-ORF-3	241206	TCGC-CTCA
pMC-ME-v2_SA-memb-mNeongreen	241207	TCGC-CTCA
pMC-ME-v2_SA-linker-P2A-mNeongreen-ORF1	241208	TCGC-CTCA
pMC-ME-v2_SA-linker-P2A-mNeongreen-ORF2	241209	TCGC-CTCA
pMC-ME-v2_SA-linker-P2A-mNeongreen-ORF3	241210	TCGC-CTCA
pMC-ME-v2_SA-EGFP-pA	241211	TCGC-CTCA
pMC-ME-v2_SA-mCherry-pA	241212	TCGC-CTCA
pMC-dest_BsaI-cmlc2:mCherry	241213	CAGT-CCAA
pMC-dest_BsaI-MCS-cloningvector forgoldengate	241214	CAGT-CCAA

File S1. Primer sequence list

Available for download at
https://journals.biologists.com/bio/article-lookup/doi/10.1242/bio.062472#supplementary-data

File S2. *foxd3* gDNA sequence

Available for download at
https://journals.biologists.com/bio/article-lookup/doi/10.1242/bio.062472#supplementary-data

File S3. *foxd3 CreER* donor vector sequence

Available for download at
https://journals.biologists.com/bio/article-lookup/doi/10.1242/bio.062472#supplementary-data

File S4. *foxd3*^{CreER} line 9 sequence

Available for download at
https://journals.biologists.com/bio/article-lookup/doi/10.1242/bio.062472#supplementary-data

File S5. *foxd3*^{CreER} line 23 sequence

Available for download at
https://journals.biologists.com/bio/article-lookup/doi/10.1242/bio.062472#supplementary-data

File S6. *hbaa1.2* gDNA sequence

Available for download at
https://journals.biologists.com/bio/article-lookup/doi/10.1242/bio.062472#supplementary-data

File S7. *hbaa1.2* ItoV donor vector sequence

Available for download at
https://journals.biologists.com/bio/article-lookup/doi/10.1242/bio.062472#supplementary-data

File S8. *hbaa1.2*^{ItoV} gDNA sequence

Available for download at
https://journals.biologists.com/bio/article-lookup/doi/10.1242/bio.062472#supplementary-data

FileS9. Tutorial file for mini-golden–mediated amino acid substitution: synthetic fragment design for *hbaa1.2* I-to-V substitution.

Available for download at
https://journals.biologists.com/bio/article-lookup/doi/10.1242/bio.062472#supplementary-data

File S10. Tutorial file for mini-golden–mediated amino acid substitution: a donor pMC vector sequence for *hbaa1.2* I-to-V substitution.

Available for download at
https://journals.biologists.com/bio/article-lookup/doi/10.1242/bio.062472#supplementary-data

Biology Open • Supplementary information

File S11. pMC-Dest-BsaI sequence

Available for download at
https://journals.biologists.com/bio/article-lookup/doi/10.1242/bio.062472#supplementary-data

File S12. pMid_acrymCherry-FRT sequence

Available for download at
https://journals.biologists.com/bio/article-lookup/doi/10.1242/bio.062472#supplementary-data

File S13. Codon_usage_change_template

Available for download at
https://journals.biologists.com/bio/article-lookup/doi/10.1242/bio.062472#supplementary-data

Biology Open • Supplementary information
